# Supplementary material for: Genotype-Phenotype Correlation of Seven Known and Novel β-Globin Gene Variants
Source: Int J Mol Sci. 2025 Sep 12;26(18):8872. doi: 10.3390/ijms26188872 (PMC12469752; doi:10.3390/ijms26188872)
Supplement: Supplementary file 1 [file ijms-26-08872-s001.zip › ijms-3829573-supplementary.pdf]

Table S1 Hb A_2 levels and β-globin gene variants found in the Thai population. Values are presented as mean ± standard deviation.

**Variants**	**Nucleotide**	**n**	**Hb A_2 (%)**
-198(A>G)	AA	86	2.8±0.2
	AG	3	2.7±0.2
	GG	0	-
CD 2 (CAT>CAC)	TT	16	2.8±0.2
	TC	43	2.8±0.2
	CC	30	2.8±0.2
IVS II-16 (G>C)	GG	16	2.8±0.2
	GC	43	2.8±0.2
	CC	30	2.8±0.2
IVS II-74 (T>G)	TT	54	2.8±0.2
	TG	33	2.8±0.2
	GG	2	2.7, 3.0
IVS II-81 (C>T)	CC	86	2.8±0.2
	CT	3	2.7±0.1
	TT	0	-
IVS II-666 (C>T)	CC	16	2.8±0.2
	CT	42	2.8±0.2
	TT	31	2.8±0.2
*233(G>C)	GG	86	2.8±0.2
	GC	3	2.7±0.1
	CC	0	-

Table S2 Similarity score related to transcription factor binding affinity of wild-type sequence compared with $\beta^{-206(C>G)}$, $\beta^{-198(A>G)}$, and $\beta^{-50(G>A)}$ using the online TFBIND program.

AC ID from TRANSFAC R.3.4	Symbol	Consensus sequence	Wild type	Alternate sequence
**$\beta^{-206(C>G)}$**				
M00109 V$CEBPB_01	C/EBPbeta (CEBPB)	RNRTKNNGMAAKNN	0.837296 (GTATGGGGCCAAGA)	<0.81
M00254 V$CAAT_01	CCAAT box	NNNRRCCAATSA	0.822367 (TGGGGCCAAGAG)	<0.78
M00008 V$SP1_01	Sp1, SP1	GRGGCRGGGW	<0.78	0.806625 (GGGGCGAAGA)
M00105 V$CDPCR3_01	CDP CR3, CUTL1	CACCRATANNTATNG	0.755502 (CCAAGAGATATATCT)	<0.75
M00126 V$GATA1_02	GATA-1	NNNNNGATANKGNN	<0.77	0.792500 (GAAGAGATATATCT)
**$\beta^{-198(A>G)}$**				
M00105 V$CDPCR3_01	CDP CR3, CUTL1	CACCRATANNTATNG	0.755502 (CCAAGAGATATATCT)	0.766505 (CCAAGAGATGTATCT)
M00128 V$GATA1_04	GATA-1	NNCWGATARNNNN	0.850797 (AAGAGATATATCTTA)	<0.81
M00076 V$GATA2_01	GATA-2	NNNGATRNNN	0.829950 (AGAGATATAT)	0.801534 (AGAGATGTAT)
M00077 V$GATA3_01	GATA-3	NNGATARNG	0.832078 (GAGATATAT)	<0.82
M00203 V$GATA_C	GATA-X	NGATAAGNMNN	0.868282 (GAGATATATCT)	0.880087 (GAGATGTATCT)
M00126 V$GATA1_02	GATA-1	NNNNNGATANKGNN	<0.77	0.779062 (AGATGTATCTTAGA)
M00203 V$GATA_C	GATA-X	NGATAAGNMNN	0.879155 (AGATATATCTT)	<0.83
M00075 V$GATA1_01	GATA-1	SNNGATNNNN	0.779862 (ATATATCTTA)	0.801086 (ATGTATCTTA)
M00076 V$GATA2_01	GATA-2	NNNGATRNNN	0.854308 (ATATATCTTA)	0.865584 (ATGTATCTTA)
**$\beta^{-50(G>A)}$**				
M00196 V$SP1_Q6	Sp1, SP1	NGGGGGCGGGGYN	0.774329 (GCAGGGAGGGCAG)	<0.75
M00008 V$SP1_01	Sp1, SP1	GRGGCRGGGW	0.845696 (CAGGGAGGGC)	0.793318 (CAGGGAGGAC)
M00127 V$GATA1_03	GATA-1	RNSNNGATAANNGN	<0.78	0.829985 (GGGAGGACAGGAGC)
M00196 V$SP1_Q6	Sp1, SP1	NGGGGGCGGGGYN	0.808259 (GGAGGGCAGGAGC)	<0.75
M00072 V$CP2_01	CP2, TFCP2	GCNMNAMCMAG	0.886792 (GCAGGAGCCAG)	0.791595 (ACAGGAGCCAG)

CEBPB = CCAAT enhancer binding protein beta, GATA-X = GATA binding site, S = C or G, W = A or T, R = A or G, Y = C or T, K = G or T, M = A or C, N = any base pair.

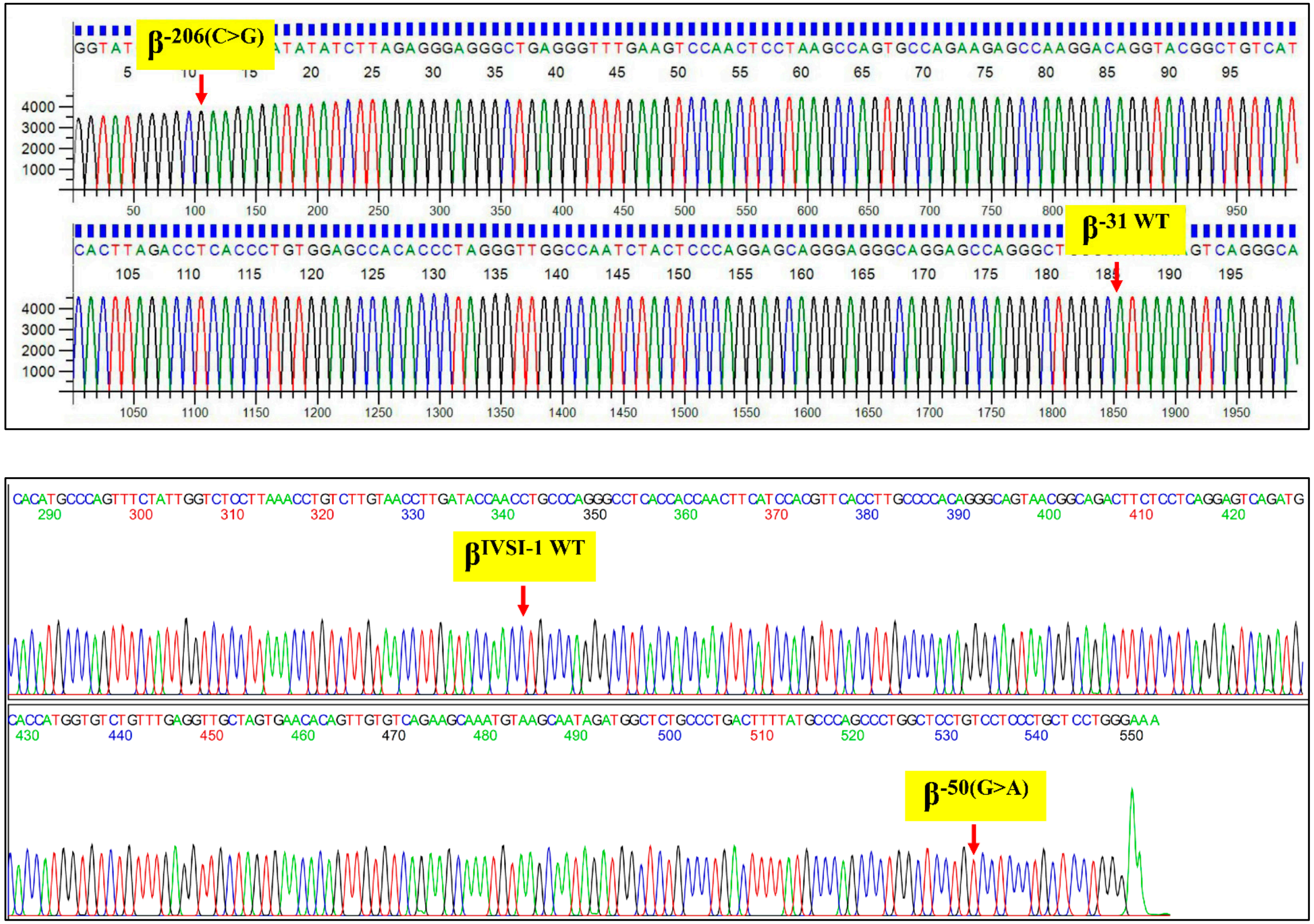

Figure S1 The representative DNA sequencing profiles of allele-specific amplification of $\beta^{-206(C>G)}$ and $\beta^{-50(G>A)}$. **A**: The $\beta^{-206(C>G)}$ specific allele has a wild-type sequence in β^{-31} in subject no 1, representing *in trans* mutation between $\beta^{-206(C>G)}$ and $\beta^{-31(A>G)}$. **B**: The $\beta^{-50(G>A)}$ specific allele has a wild-type sequence in β^{IVSI-1} in subject no 21, representing *in trans* mutation between $\beta^{-50(G>A)}$ and $\beta^{IVSI-1(G>T)}$.
